# Porokeratotic adnexal ostial nevus: A paradigm of cutaneous mosaicism

**DOI:** 10.1002/ccr3.5728

**Published:** 2022-04-12

**Authors:** Lisa Kiely, Sarah Ni Mhaolcatha, Jim Fitzgibbon, Lesley‐Ann Murphy, Cathal O’Connor

**Affiliations:** ^1^ 8795 Department of Dermatology South Infirmary Victoria University Hospital Cork Ireland; ^2^ 57983 Department of Pathology Cork University Hospital Cork Ireland; ^3^ 8795 Department of Paediatrics and Child Health University College Cork Cork Ireland

**Keywords:** mosaicism, PAON, PEODDN, porokeratosis

## Abstract

Porokeratotic adnexal ostial nevus (PAON) is a term encompassing porokeratotic eccrine ostial and dermal duct naevus (PEODDN) and porokeratotic eccrine and hair follicle naevus (PEHFN). We present the case of a 7‐year‐old girl who presented with hyperkeratotic verrucous papules in a blaschkolinear distribution on the sole of her left foot.

## Graphical abstract

Porokeratotic adnexal ostial nevus (PAON) encompasses porokeratotic eccrine ostial and dermal duct nevus and porokeratotic eccrine and hair follicle nevus. Somatic mutations in GJB2 have been identified as causative in PAON, representing a mosaic form of keratosis ichthyosis deafness (KID) syndrome.

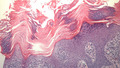

## 1 INTRODUCTION

Porokeratotic adnexal ostial nevus (PAON) is a term that encompasses porokeratotic eccrine ostial and dermal duct nevus (PEODDN) and porokeratotic eccrine and hair follicle nevus (PEHFN).^1^ PAON is a rare adnexal hamartoma characterized by the presence of a cornoid lamella exclusively overlying eccrine acrosyringia (the subtype known as PEODDN) or over both eccrine acrosyringia and hair follicles (the subtype known as PEHFN).

## 2 CASE REPORT

**FIGURE 1:**
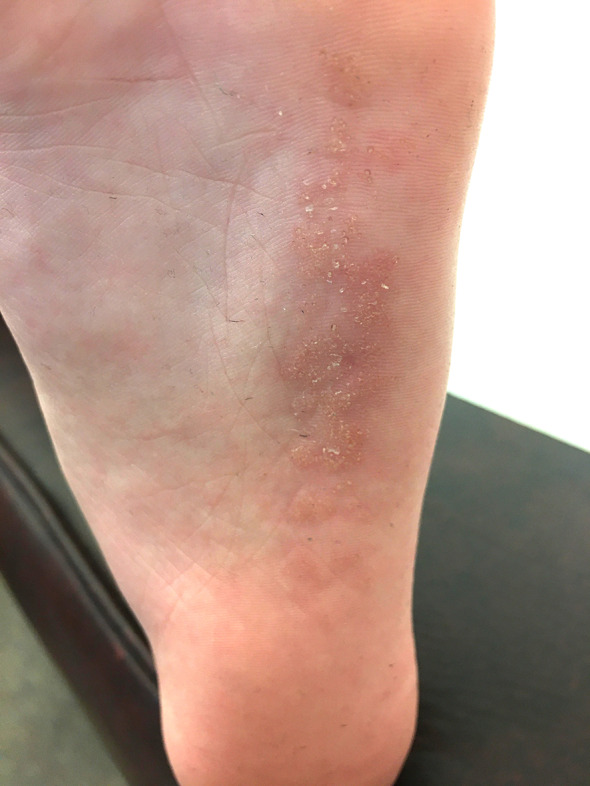
Close‐up view of the left plantar foot, showing hyperkeratotic verrucous papules in a blaschkolinear distribution

A 7‐year‐old girl presented with hyperkeratotic verrucous papules in a blaschkolinear distribution on the sole of her left foot. The lesions were congenital and asymptomatic. She had a family history of atopic dermatitis but no significant past medical history. Prior to presentation, the lesions were treated with topical antifungals and salicylic acid, with no conferred benefit. On examination, there were unilateral hyperkeratotic papules coalescing into plaques in a linear configuration following lines of Blashko on the lateral border of the left sole (Figure 1).

**FIGURE 2:**
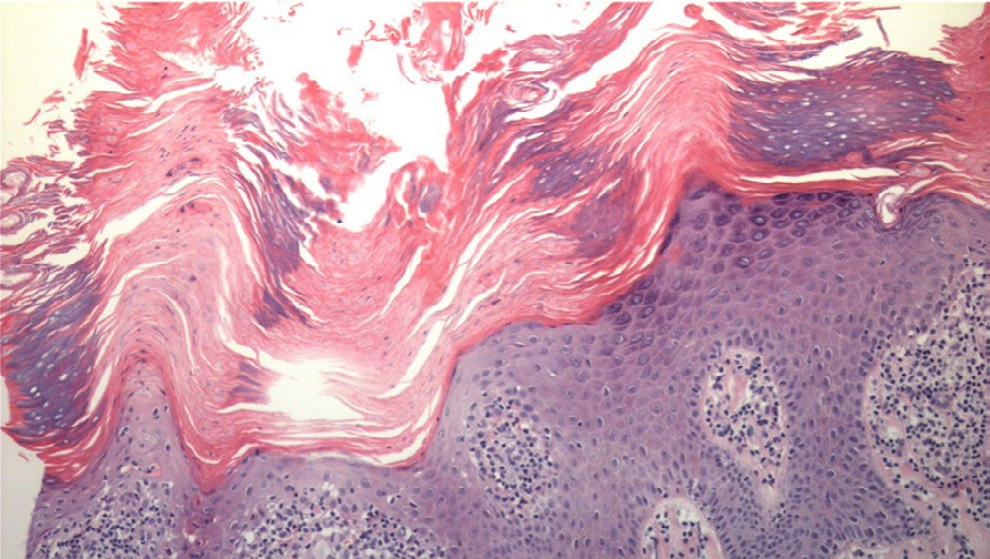
Microscopic sections of acral skin show papillary epidermal hyperplasia with alternating parakeratosis of the stratum corneum and orthokeratosis. Columns of parakeratosis in the troughs are associated with scant dyskeratotic keratinocytes and loss of the granular cell layer beneath columns of parakeratosis, reminiscent of a cornoid lamella

Histopathological analysis showed columns of parakeratosis associated with scant dyskeratotic keratinocytes in the upper granular layer reminiscent of cornoid lamellae (Figure 2). A deep infiltrate involving sweat ducts was absent.

## 3 DISCUSSION

Porokeratotic adnexal ostial nevus is characterized by small discrete scaly papules arranged linearly, which may coalesce into plaques. Usually unilateral, most common sites are the palm or sole, with occasional extension onto the dorsae of the hands and feet, although more generalized eruptions have been reported.^2^ Lesions are typically asymptomatic. The male to female ratio is almost 1:1.^2^ PEODDN is usually a congenital phenomenon; however, up to 26% of cases are reported to be late‐onset, with some cases noted to develop following puberty.^3^ PEODDN usually becomes more warty in appearance over time.

Porokeratotic adnexal ostial nevus is a disorder of the intraepidermal eccrine duct (acrosyringium). Histopathology is diagnostic, characterized by parakeratotic columns within an epidermal invagination appearing as cornoid lamellae. Vacuolated and dyskeratotic keratinocytes are often present, and there may be loss of the granular layer.^4^ Somatic mutations in GJB2 have been identified as causative in PAON, representing a mosaic form of keratosis ichthyosis deafness (KID) syndrome. GJB2 encodes the gap junction protein connexin26 (Cx26), which permits intercellular ion and macromolecule flux.^5^ Previously reported cases of PAON have described coexisting problems like seizures, hemiparesis, scoliosis, deafness, developmental delay, palmoplantar keratoderma, psoriasis, hyperthyroidism, polyneuropathy, breast hypoplasia, and KID.^2^

The differential diagnosis of PAON includes variants of porokeratosis, nevus comedonicus, epidermal nevi, viral warts, or porokeratoma. Linear or punctate porokeratosis lacks the combination of cornoid lamellae with acrosyringia or hair follicle ostia, the eccrine duct hyperplasia, and the thicker epidermal invagination. Nevus comedonicus and epidermal nevus (or inflammatory linear verrucous epidermal nevus) can rarely present with porokeratosis‐like features but are not related to eccrine ostia or hair follicle. Focal hyperparakeratosis, koilocytes, and hemorrhagic exudates are seen in viral warts but not in PAON. In this case, eccrine duct involvement was not clear on histology, but a diagnosis of PAON was favored following clinicopathological correlation.

Current treatment options are of limited efficacy. Reported therapies include topical corticosteroids, topical salicylic acid, and topical retinoids such as tazarotene, photodynamic therapy, and carbon dioxide laser.^2^, ^3^ While the lesions tend to be refractory to treatment, they are usually asymptomatic and may not require intervention. The patient and parents in this case declined further intervention due to asymptomatic nature of the nevus.

This case highlights a typical presentation of a rare condition whose clinical features are often initially subtle and can be easily overlooked. There is evidence that PAON is a manifestation of somatic mosaicism for KID syndrome and, although never previously reported, there is a theoretical risk of transmission of systemic disease to offspring.

## CONFLICT OF INTEREST

None.

## AUTHOR CONTRIBUTION

COC involved in conception, analysis, writing, and manuscript review. LK involved in analysis and writing. LAM involved in conception and manuscript review. SNM and JF involved in histological review and manuscript review.

## CONSENT

Written informed consent was obtained from the patient to publish this report in accordance with the journal's patient consent policy.
